# Temporal trends, disparities, and ARIMA forecasts of mortality among U.S. adults with coexisting hematologic malignancy and heart failure, 1999–2023, with projections to 2033

**DOI:** 10.3389/fonc.2026.1818278

**Published:** 2026-04-22

**Authors:** Yue Zhang, Ying Tian

**Affiliations:** 1 Department of Scientific Research, Beijing Haidian Hospital, Beijing, China; 2 Department of Hematology, Beijing Chao-Yang Hospital, Capital Medical University, Beijing, China

**Keywords:** ARIMA forecasting, cardio-oncology, health disparities, heart failure, hematologic malignancies, Joinpoint regression, mortality trends

## Abstract

**Background:**

Hematologic malignancies (HMs) and heart failure (HF) increasingly coexist as populations age, yet population-level mortality patterns in adults with both conditions remain poorly defined. We characterized long-term temporal trends and demographic disparities in HM–HF mortality and forecasted future rates through predictive modeling.

**Methods:**

We analyzed U.S. multiple cause-of-death data (1999–2023) for adults aged ≥45 years with both HMs and HF listed on death certificates. Age-adjusted mortality rates (AAMRs) were calculated per 100,000 population. Joinpoint regression was used to identify changes in temporal trends, with analyses stratified by demographics, HM subtype, and geography. Autoregressive integrated moving average (ARIMA) modeling was applied to project mortality trends through 2033.

**Results:**

Among 119,147 deaths, the overall AAMR was 3.96 per 100,000 (95% CI, 3.85–4.07). Joinpoint analysis identified four phases: a significant decline from 1999 to 2013 (annual percent change [APC] −1.97%), a plateau from 2013 to 2018, a sharp increase from 2018 to 2021 (APC + 8.35%), and subsequent stabilization from 2021 to 2023. Mortality was 85% higher in males than females (5.47 vs. 2.95 per 100,000). Rates increased markedly with age, with adults aged ≥85 years showing the steepest rise after 2014 (APC + 5.75%). Non-Hispanic Black and non-Hispanic White populations had the highest absolute AAMRs. All HM subtypes demonstrated a similar decline–rebound pattern, with subtype-specific differences in the timing of inflection points. ARIMA projections suggested a gradual decline from 4.78 per 100,000 in 2024 to 3.77 per 100,000 by 2033.

**Conclusions:**

HM–HF mortality declined through 2013 but reversed in the late 2010s, with pronounced increases during 2018–2021 followed by recent stabilization. Persistent disparities by sex, age, race/ethnicity, and geography highlight the need for targeted interventions and strengthened cardio-oncology capacity for this high-risk population.

## 1 Introduction

Hematologic malignancies (HMs) and heart failure (HF) increasingly co-occur in older adults due to population aging and improved cancer survival rates, creating a complex clinical phenotype associated with substantial mortality burden. Both conditions disproportionately affect older adults: cancer incidence and mortality peak in later life, while HF prevalence and adverse outcomes increase markedly with advancing age, underscoring the clinical importance of their intersection (1, 2).

The pathophysiology underlying concurrent HM-HF reflects multiple converging mechanisms. Shared risk factors include cardiometabolic comorbidities, chronic systemic inflammation, and increased susceptibility to infections. In addition, cardiotoxicity from cancer treatments represents a major contributor to cardiovascular morbidity. Cardio-oncology guidelines identify anthracyclines as well-established cardiotoxic agents, while newer targeted therapies pose emerging cardiovascular risks (3). In hematologic cancers specifically, proteasome inhibitors—particularly carfilzomib—increase risks of hypertension and heart failure, while Bruton tyrosine kinase inhibitors such as ibrutinib are associated with atrial and ventricular arrhythmias, hypertension, and HF (4, 5).

The therapeutic landscape for both disease domains has evolved substantially over the past 25 years. Hematologic oncology has been transformed by the introduction of rituximab and other monoclonal antibodies in the early 2000s, proteasome inhibitors and immunomodulatory drugs for multiple myeloma (MM), and recent advances including CAR-T cell therapy and novel targeted agents (6–8). Simultaneously, HF management has experienced significant advances, including angiotensin receptor-neprilysin inhibitors (ARNIs), which demonstrated superior outcomes compared to traditional ACE inhibitors (9), and sodium-glucose co-transporter 2 (SGLT2) inhibitors, which have shown cardiovascular benefits in HF patients regardless of diabetes status (10). These pharmacological innovations have been incorporated into evidence-based guidelines emphasizing personalized, multidisciplinary management approaches (11).

These parallel therapeutic advances create complex interactions affecting long-term mortality trajectories. While improved HM treatments may increase survival and subsequent exposure to treatment-related cardiovascular complications, concurrent advances in HF management may mitigate some cardiotoxic effects and improve overall outcomes in this vulnerable population.

Surveillance data reveal significant demographic disparities in both HMs and HF outcomes that may compound when these conditions co-occur. Racial and ethnic minorities experience substantial disparities in HF outcomes, including higher mortality rates and more advanced disease at presentation (12). Cancer outcomes similarly vary significantly across demographic groups, as documented in national surveillance data (13). Geographic location affects acute cardiovascular care outcomes, with rural populations experiencing different mortality patterns compared to urban populations (14). Whether these established disparities translate to differential mortality patterns in the HM-HF population remains unclear.

Recent population-level research has begun to examine the intersection of cancer and HF mortality using national death certificate data. In particular, Qazi et al. reported temporal trends in cancer- and HF-related mortality in the adult U.S. population using the CDC WONDER database from 1999 to 2020, underscoring the growing public health relevance of this overlap (15). However, that analysis addressed cancer more broadly and was not focused specifically on hematologic malignancies, did not examine major HM subtype-specific patterns, and did not extend into the most recent post-2020 period. Prior research has also largely emphasized disease-specific clinical cohorts, such as studies of lymphoma subtypes in patients with preexisting HF (16, 17). Accordingly, a dedicated national analysis of mortality involving both HM and HF, with detailed demographic and geographic stratification and contemporary trend assessment, remains needed.

Understanding HM-HF mortality trends has significant clinical and public health implications. As cancer survivorship improves, the absolute number of individuals at risk for concurrent HMs and HF continues to grow (18). Healthcare resource planning and quality improvement initiatives require accurate mortality surveillance to identify high-risk subgroups and periods of vulnerability.

Traditional epidemiological surveillance provides valuable retrospective insights but offers limited guidance for future healthcare planning and resource allocation. Predictive modeling through time series analysis has emerged as a critical tool for projecting mortality trends and informing proactive healthcare strategies. Autoregressive Integrated Moving Average (ARIMA) models demonstrate particular utility in epidemiological forecasting through their capacity to handle non-stationary time series data and capture complex temporal dependencies in mortality patterns (19, 20). Recent ARIMA applications in healthcare forecasting have yielded essential insights for public health planning during periods of epidemiological transition (21, 22).

We conducted a comprehensive analysis of U.S. mortality data from 1999 through 2023 to characterize temporal trends and demographic disparities in deaths among adults aged ≥45 years with concurrent HMs and HF. Joinpoint regression methodology identified periods of significant change in mortality patterns and examined variation across sex, age, race/ethnicity, HM subtype, and geographic characteristics. ARIMA-based predictive modeling was employed to forecast mortality trends through 2033, providing prospective insights for healthcare planning and resource allocation in this vulnerable population.

## 2 Methods

### 2.1 Study design and data source

We performed a retrospective, population-based analysis of U.S. mortality using the Centers for Disease Control and Prevention (CDC) Wide-Ranging Online Data for Epidemiologic Research (WONDER) Multiple Cause-of-Death (MCOD) Public Use files for calendar years 1999–2023 (23). These files include all death certificates filed in the 50 U.S. states and the District of Columbia and provide information on underlying and contributing causes of death coded according to the International Classification of Diseases, Tenth Revision (ICD-10), along with decedent demographics and geographic characteristics.

### 2.2 Study population

We included all decedents aged ≥45 years who had both a HM and HF listed anywhere on their death certificate (as either underlying or contributing causes of death). The ≥45-year threshold was prespecified to target the age range in which HMs and HF are most prevalent and to ensure stable rate estimates in stratified analyses. HMs were identified using ICD-10 codes C81-C96, with prespecified subgroups: Non-Hodgkin lymphoma (NHL, C82-C85), MM (C90.0), chronic lymphocytic leukemia (CLL, C91.1), acute myeloid leukemia (AML, C92.0), and chronic myeloid leukemia (CML, C92.1). HF was defined using ICD-10 codes I50.x and hypertensive heart/renal disease with HF (I11.0, I13.0, I13.2). These categories were selected based on their epidemiologic importance and high prevalence in middle-aged and older U.S. adults.

### 2.3 Demographic and geographic variables

Race and ethnicity were classified according to U.S. Office of Management and Budget standards as non-Hispanic (NH) White, NH Black or African American, NH Other (including American Indian/Alaska Native and Asian/Pacific Islander), and Hispanic/Latino (24). Annual population denominators by sex, age group, race, and Hispanic/Latino ethnicity were obtained from U.S. Census Bureau intercensal and postcensal estimates (25). Geographic variables included urban–rural status using the 2013 National Center for Health Statistics Urban–Rural Classification Scheme (large metropolitan areas [≥1,000,000 population], medium/small metropolitan areas [50,000–999,999], and rural areas [<50,000]) (26), and U.S. Census regions (Northeast, Midwest, South, and West) (27).

### 2.4 Statistical analysis

Crude mortality rates (CMRs) and age-adjusted mortality rates (AAMRs) were calculated per 100,000 persons with 95% confidence intervals, standardized to the 2000 U.S. standard population using the direct method. Joinpoint regression (Joinpoint Regression Program, version 5.1.0.0; National Cancer Institute) was applied to characterize changes in mortality trends over time. This method identifies statistically significant changes (“joinpoints”) in temporal trajectories and calculates annual percent changes (APCs) for each segment and average annual percent changes (AAPCs) over the entire period (28, 29).

Given 25 annual data points, up to four potential joinpoints were allowed, following established recommendations for joinpoint analysis. Parallelism tests were performed to compare subgroup trends, with significant interaction *P*-values indicating that average annual percent changes differed between groups.

All analyses were conducted using Joinpoint (version 5.1.0.0) (30) and R software (version 4.4.2; R Foundation for Statistical Computing, Vienna, Austria). Two-sided *P* < 0.05 was considered statistically significant.

### 2.5 Predictive time series analysis

An Autoregressive Integrated Moving Average (ARIMA) model was employed to forecast mortality rates through 2033. The optimal ARIMA(p,d,q) specification was identified using automated selection (auto.arima) based on information criteria optimization (31). Model validation included comprehensive residual diagnostics and the Ljung-Box test to verify absence of serial correlation (32, 33). Ten-year projections were generated with 95% confidence intervals to quantify forecast uncertainty.

### 2.6 Ethical considerations

Because the CDC WONDER database is publicly available and fully de-identified, institutional review board approval and informed consent were not required. The study was conducted in accordance with STROBE guidelines for observational research.

## 3 Results

### 3.1 Overall mortality trends

Between 1999 and 2023, 119,147 deaths among U.S. adults aged ≥45 years listed both HM and HF on the death certificate. The overall AAMR was 3.96 per 100,000 (95% CI: 3.85–4.07), with a modest but statistically significant long-term increase (AAPC + 0.51%; 95% CI: 0.33–0.67; **Table 1**).

**Table 1:** Characteristics of decedents with hematologic malignancy (HM) and heart failure (HF) in the United States, 1999-2023.

Characteristic	Deaths (n)	AAMR per 100,000 (95% CI)	AAPC (95% CI)
Overall	119,147	3.96 (3.85, 4.07)	0.51 (0.33, 0.67)
Sex			
Male	66,492	5.47 (5.26, 5.69)	0.80 (0.54, 1.09)
Female	52,655	2.95 (2.82, 3.07)	-0.05 (-0.25, 0.15)
Age group (years)			
45-54	2,378	0.23 (0.18, 0.28)	1.17 (0.10, 2.26)
55-64	8,018	0.90 (0.80, 1.00)	0.35 (-0.20, 0.94)
65-74	21,930	3.57 (3.33, 3.81)	-0.21 (-0.73, 0.69)
75-84	43,849	12.41 (11.83, 12.99)	0.51 (0.27, 0.76)
85+	42,972	30.96 (29.49, 32.44)	1.16 (0.78, 1.57)
Race/ethnicity			
NH White	101,739	4.22 (4.09, 4.35)	0.74 (0.55, 0.90)
NH Black	9,925	3.69 (3.32, 4.07)	0.87 (0.59, 1.18)
Hispanic/Latino	4,942	2.27 (1.93, 2.62)	0.27 (-0.51, 1.20)
NH Other (AI/AN, Asian/PI)	2,351	1.78 (1.40, 2.22)	-0.06 (-1.26, 1.41)
Hematologic malignancy subtype			
Non-Hodgkin lymphoma (NHL)	42,353	1.43 (1.36, 1.49)	0.40 (0.18, 0.68)
Multiple myeloma (MM)	26,423	0.86 (0.82, 0.93)	0.97 (0.63, 1.29)
Chronic lymphocytic leukemia (CLL)	19,897	0.66 (0.62, 0.71)	0.79 (0.43, 1.19)
Acute myeloid leukemia (AML)	8,665	0.27 (0.24, 0.30)	3.01 (2.47, 3.66)
Chronic myeloid leukemia (CML)	4,485	0.14 (0.12, 0.16)	1.71 (0.78, 2.79)
Geographic region			
Northeast	21,824	3.67 (3.42, 3.91)	0.07 (-0.26, 0.40)
Midwest	31,230	4.62 (4.36, 4.88)	0.71 (0.47, 0.97)
South	39,896	3.64 (3.46, 3.82)	0.94 (0.66, 1.31)
West	26,197	4.07 (3.82, 4.33)	0.86 (0.60, 1.13)
Urban–rural status			
Large metropolitan	45,068	3.48 (3.32, 3.63)	0.05 (-0.19, 0.31)
Medium/small metropolitan	31,665	3.96 (3.76, 4.17)	0.40 (0.08, 0.74)
Rural	20,885	4.59 (4.29, 4.88)	0.29 (0.03, 0.54)

AAMR, age-adjusted mortality rate; AAPC, average annual percent change; CI, confidence interval; HM, hematologic malignancy; HF, heart failure; NH, non-Hispanic; AI/AN, American Indian/Alaska Native; PI, Pacific Islander.

Death counts may not sum to totals because of overlapping categories. Urban–rural classification is based on the NCHS Urban-Rural Classification Scheme for Counties.

**Figure 1:**
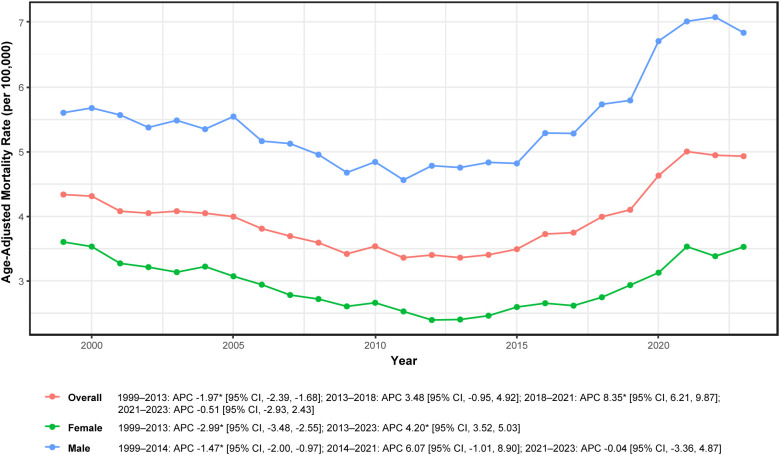
Overall and sex-specific mortality trends in adults aged ≥45 years with hematologic malignancy and heart failure, United States, 1999-2023. APC, annual percent change; * indicates *P* < 0.05 by Joinpoint. Rates per 100,000; age-adjusted to the 2000 U.S. standard population.

As illustrated in **Figure 1**, four distinct temporal segments were identified: a significant decline during 1999–2013 (APC −1.97%; 95% CI: −2.39 to −1.68), a non-significant increase during 2013–2018 (APC + 3.48%; 95% CI: −0.95 to 4.92), a marked and significant surge during 2018–2021 (APC + 8.35%; 95% CI: 6.21–9.87), and no significant change during 2021–2023 (APC −0.51%; 95% CI: −2.93 to 2.43). These segmented patterns align with annual AAMRs reported in **Supplementary Table 1**, which declined from 4.34 per 100,000 in 1999 to 3.36 in 2013, subsequently increased to 5.00 in 2021, and stabilized at 4.93 in 2023. Complete joinpoint results, including APCs, 95% confidence intervals, and *P* values for each segment, are provided in **Supplementary Table 2**.

### 3.2 Sex-specific mortality trends

Male deaths accounted for 66,492 cases (55.8%) and exhibited higher mortality rates than females (AAMR 5.47 vs. 2.95 per 100,000; 95% CIs: 5.26–5.69 and 2.82–3.07, respectively; **Table 1**). Over the 25-year period, male mortality increased significantly (AAPC + 0.80%; 95% CI: 0.54–1.09), whereas female mortality showed no significant overall change (AAPC −0.05%; 95% CI: −0.25 to 0.15).

Annual AAMRs reflected this divergent pattern: among males, rates declined from 5.60 in 1999 to 4.75 in 2013, then rose to 7.01 in 2021 and 6.84 in 2023; among females, rates decreased from 3.60 in 1999 to 2.40 in 2013, then increased to 3.53 in both 2021 and 2023 (per 100,000; **Supplementary Table 1**).

Joinpoint analysis (**Figure 1**; **Supplementary Table 2**) identified distinct temporal segments for each sex. For males: a significant decline during 1999–2014 (APC −1.47%; 95% CI: −2.00 to −0.97), a non-significant increase during 2014–2021 (APC + 6.07%; 95% CI: −1.01 to 8.90), and no significant change during 2021–2023 (APC −0.04%; 95% CI: −3.36 to 4.87). For females: a significant decline during 1999–2013 (APC −2.99%; 95% CI: −3.48 to −2.55) followed by a significant increase during 2013–2023 (APC + 4.20%; 95% CI: 3.52–5.03). Overall, sex-specific trajectories paralleled those of the total population, characterized by prolonged pre-2013 decline, post-2013 rebound, and sustained increases through the early 2020s.

### 3.3 Age-specific mortality trends

**Figure 2:**
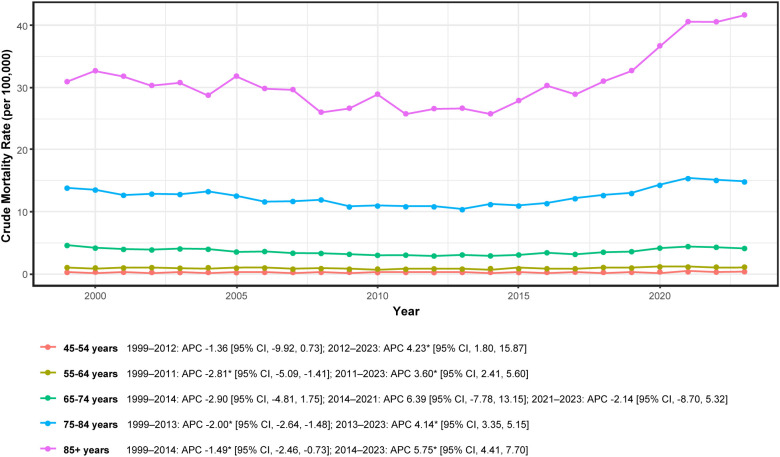
Age-specific mortality trends in adults aged ≥45 years with hematologic malignancy and heart failure, United States, 1999-2023. APC, annual percent change; * indicates *P* < 0.05 by Joinpoint. Rates per 100,000.

Age-specific mortality rates increased monotonically with advancing age, with the oldest cohorts bearing the greatest absolute burden (**Figure 2**; **Table 1**). Age-specific CMRs per 100,000 are detailed in **Supplementary Table 3**. Joinpoint analysis (**Supplementary Table 2**) identified a common temporal inflection point in the early-to-mid-2010s across most age strata.

Among adults aged 45–54 years, mortality declined modestly during 1999–2012 (APC −1.36%; 95% CI: −9.92 to 0.73; not significant) and subsequently increased significantly during 2012–2023 (APC + 4.23%; 95% CI: 1.80 to 15.87). For adults aged 55–64 years, a significant decline during 1999–2011 (APC −2.81%; 95% CI: −5.09 to −1.41) was followed by a significant increase during 2011–2023 (APC + 3.60%; 95% CI: 2.41 to 5.60).

Among adults aged 65–74 years, three temporal segments were identified: 1999–2014 (APC −2.90%; 95% CI: −4.81 to 1.75), 2014–2021 (APC + 6.39%; 95% CI: −7.78 to 13.15), and 2021–2023 (APC −2.14%; 95% CI: −8.70 to 5.32). None of these segments achieved statistical significance, indicating relative stability despite annual fluctuations.

In contrast, older age groups demonstrated clear trend reversals. Adults aged 75–84 years exhibited a significant decline during 1999–2013 (APC −2.00%; 95% CI: −2.64 to −1.48) followed by a significant increase during 2013–2023 (APC + 4.14%; 95% CI: 3.35 to 5.15). Similarly, adults aged ≥85 years experienced declining rates during 1999–2014 (APC −1.49%; 95% CI: −2.46 to −0.73) before rising significantly during 2014–2023 (APC + 5.75%; 95% CI: 4.41 to 7.70; **Figure 2**; **Supplementary Table 3**).

### 3.4 Race/ethnicity–specific mortality trends

**Figure 3:**
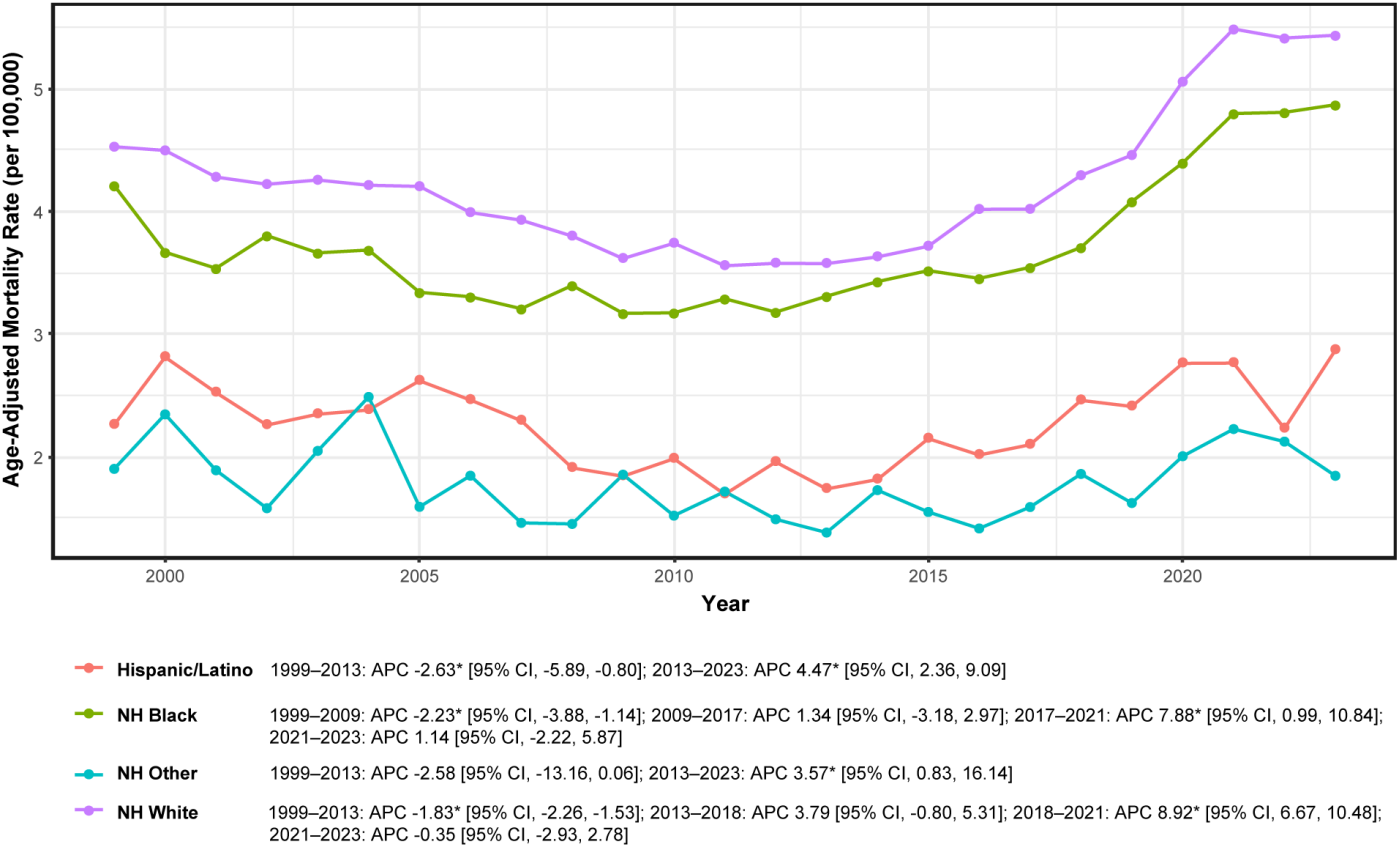
Race/ethnicity-specific mortality trends in adults aged ≥45 years with hematologic malignancy and heart failure, United States, 1999–2023. APC, annual percent change; NH, non-Hispanic; * indicates *P* < 0.05 by Joinpoint. Rates per 100,000; age-adjusted to the 2000 U.S. standard population.

NH White and NH Black populations exhibited higher mortality rates than Hispanic/Latino and NH Other groups (**Table 1**; **Supplementary Table 4**). Annual race-specific AAMRs (per 100,000) are detailed in **Supplementary Table 4**. Joinpoint analysis (**Figure 3**; **Supplementary Table 2**) revealed a consistent pattern of pre-2013 decline followed by post-2013 increases across all racial/ethnic groups, with group-specific variations in timing and magnitude.

Among Hispanic/Latino individuals, rates declined significantly during 1999–2013 (APC −2.63%; 95% CI: −5.89 to −0.80) and subsequently increased significantly during 2013–2023 (APC + 4.47%; 95% CI: 2.36 to 9.09). NH Black individuals experienced a more complex trajectory: significant decline during 1999–2009 (APC −2.23%; 95% CI: −3.88 to −1.14), a non-significant stabilization period during 2009–2017 (APC + 1.34%; 95% CI: −3.18 to 2.97), a significant surge during 2017–2021 (APC + 7.88%; 95% CI: 0.99 to 10.84), and no significant change during 2021–2023 (APC + 1.14%; 95% CI: −2.22 to 5.87).

The NH Other group demonstrated a non-significant decline during 1999–2013 (APC −2.58%; 95% CI: −13.16 to 0.06) followed by a significant increase during 2013–2023 (APC + 3.57%; 95% CI: 0.83 to 16.14). NH White individuals exhibited the most segmented pattern: significant decline during 1999–2013 (APC −1.83%; 95% CI: −2.26 to −1.53), non-significant increase during 2013–2018 (APC + 3.79%; 95% CI: −0.80 to 5.31), significant surge during 2018–2021 (APC + 8.92%; 95% CI: 6.67 to 10.48), and no significant change during 2021–2023 (APC −0.35%; 95% CI: −2.93 to 2.78; **Figure 3**; **Supplementary Table 4**).

### 3.5 HM subtype–specific mortality trends

**Figure 4:**
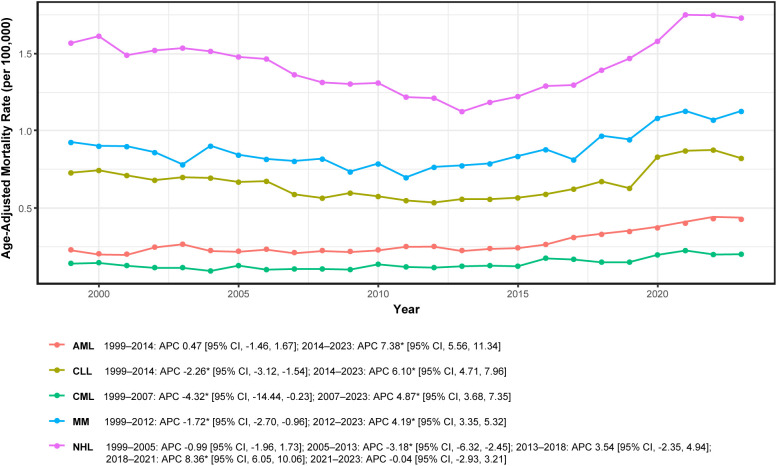
Subtype-specific mortality trends in adults aged ≥45 years with hematologic malignancy and heart failure, United States, 1999–2023. APC, annual percent change; AML, acute myeloid leukemia; CLL, chronic lymphocytic leukemia; CML, chronic myeloid leukemia; MM, multiple myeloma; NHL, non-Hodgkin lymphoma; * indicates *P* < 0.05 by Joinpoint. Rates per 100,000; age-adjusted to the 2000 U.S. standard population.

Subtype-specific mortality trajectories were heterogeneous yet demonstrated a common pattern of early decline followed by acceleration beginning in the mid-2010s (**Figure 4**; **Table 1**). Annual subtype-specific AAMRs (per 100,000) are reported in **Supplementary Table 5**, with corresponding segmented estimates provided in **Supplementary Table 2**.

AML rates remained stable during 1999–2014 (APC 0.47%; 95% CI: −1.46 to 1.67) and subsequently increased significantly during 2014–2023 (APC + 7.38%; 95% CI: 5.56 to 11.34). CLL declined significantly during 1999–2014 (APC −2.26%; 95% CI: −3.12 to −1.54) and increased thereafter during 2014–2023 (APC + 6.10%; 95% CI: 4.71 to 7.96). CML demonstrated an early significant decline during 1999–2007 (APC −4.32%; 95% CI: −14.44 to −0.23) followed by sustained growth during 2007–2023 (APC + 4.87%; 95% CI: 3.68 to 7.35).

MM rates decreased significantly during 1999–2012 (APC −1.72%; 95% CI: −2.70 to −0.96) and subsequently increased significantly during 2012–2023 (APC + 4.19%; 95% CI: 3.35 to 5.32). NHL exhibited the most complex multisegment trajectory: non-significant decline during 1999–2005 (APC −0.99%; 95% CI: −1.96 to 1.73), significant decline during 2005–2013 (APC −3.18%; 95% CI: −6.32 to −2.45), non-significant increase during 2013–2018 (APC + 3.54%; 95% CI: −2.35 to 4.94), significant surge during 2018–2021 (APC + 8.36%; 95% CI: 6.05 to 10.06), and no significant change during 2021–2023 (APC −0.04%; 95% CI: −2.93 to 3.21).

Collectively, these subtype-specific trends mirror the overall temporal pattern—prolonged decline through the early-to-mid-2010s followed by post-2013 rebound and recent increases—while demonstrating considerable variation in onset timing and magnitude across AML, CLL, CML, MM, and NHL subtypes (**Figure 4**; **Supplementary Tables 2**, **5**).

### 3.6 Geographic variation by U.S. census region

**Figure 5:**
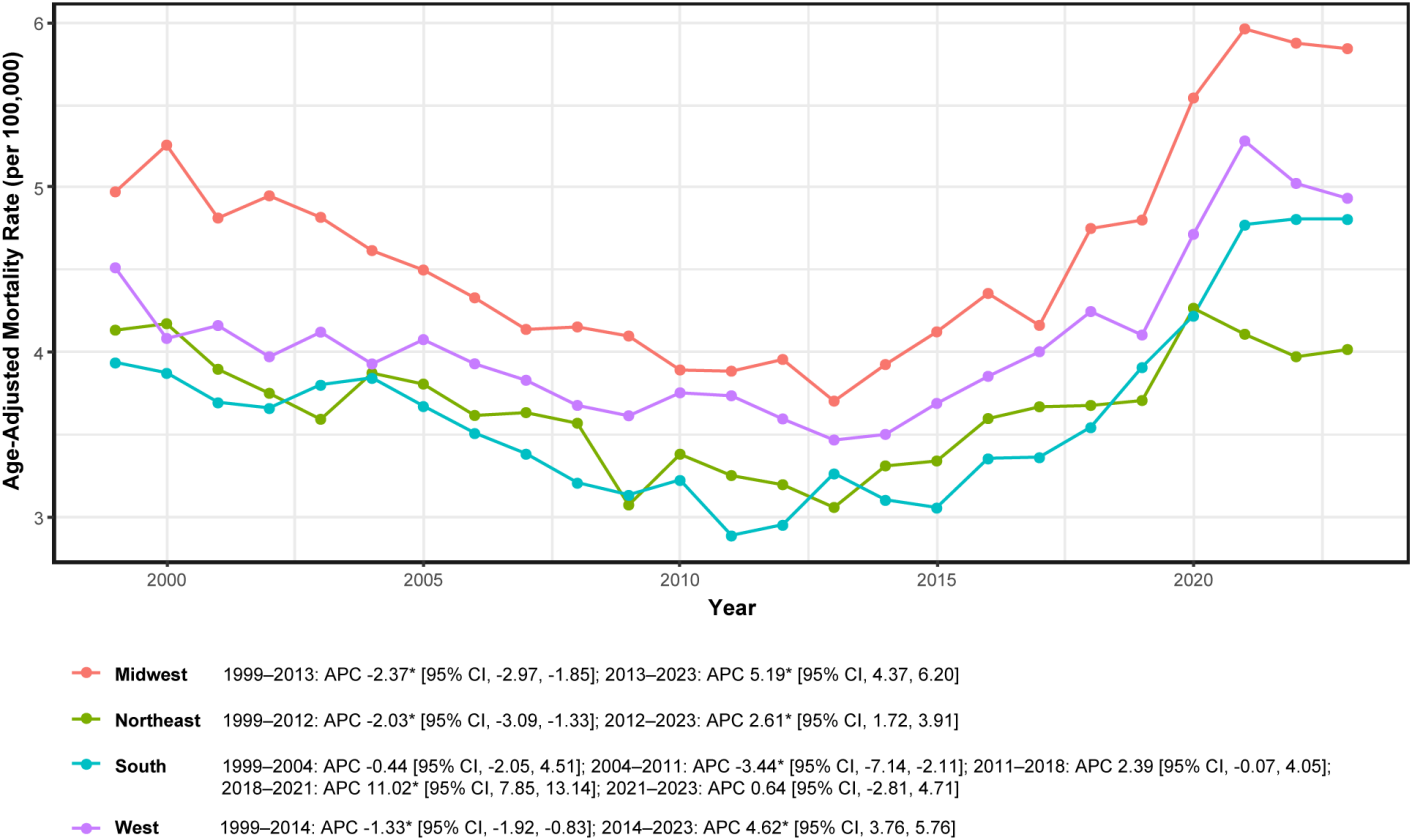
Age-adjusted mortality by U.S. Census region in adults aged ≥45 years with hematologic malignancy and heart failure, United States, 1999-2023. *Note:* APC, annual percent change; * indicates *P* < 0.05 by Joinpoint. Rates per 100,000; age-adjusted to the 2000 U.S. standard population.

Regional mortality patterns demonstrated broad concordance, characterized by prolonged decline through the early 2010s followed by sustained increases, with regional variations in timing and magnitude (**Figure 5**; **Table 1**). Joinpoint analysis (**Supplementary Table 2**) revealed distinct temporal trajectories for each region.

The Midwest exhibited a significant decline during 1999–2013 (APC −2.37%; 95% CI: −2.97 to −1.85) followed by a significant increase during 2013–2023 (APC + 5.19%; 95% CI: 4.37 to 6.20). The Northeast demonstrated a similar biphasic pattern with significant decline during 1999–2012 (APC −2.03%; 95% CI: −3.09 to −1.33) and subsequent increase during 2012–2023 (APC + 2.61%; 95% CI: 1.72 to 3.91).

The South demonstrated the most complex multisegment trajectory: non-significant change during 1999–2004 (APC −0.44%; 95% CI: −2.05 to 4.51), significant decline during 2004–2011 (APC −3.44%; 95% CI: −7.14 to −2.11), non-significant increase during 2011–2018 (APC + 2.39%; 95% CI: −0.07 to 4.05), marked increase during 2018–2021 (APC + 11.02%; 95% CI: 7.85 to 13.14), and no significant change during 2021–2023 (APC + 0.64%; 95% CI: −2.81 to 4.71). The West showed significant decline during 1999–2014 (APC −1.33%; 95% CI: −1.92 to −0.83) followed by significant increase during 2014–2023 (APC + 4.62%; 95% CI: 3.76 to 5.76).

Annual region-specific AAMRs (per 100,000) are detailed in **Supplementary Table 6** and align with these segmented trends, highlighting post-2013 rebound across all regions and a particularly pronounced 2018–2021 increase in the South.

### 3.7 Urban–rural variation

**Figure 6:**
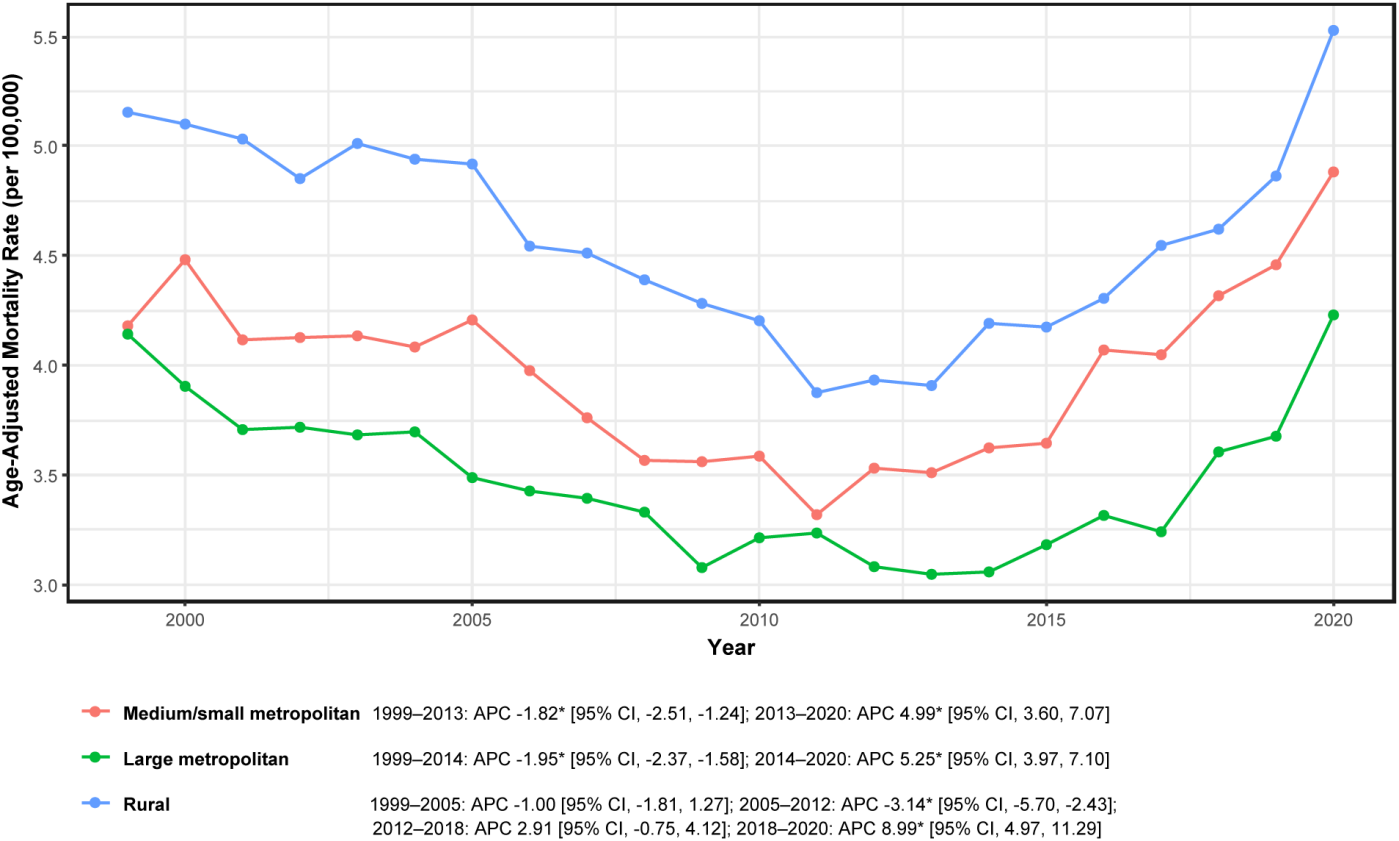
Age-adjusted mortality by urban–rural category in adults aged ≥45 years with hematologic malignancy and heart failure, United States, 1999–2020. APC, annual percent change; * indicates *P* < 0.05 by Joinpoint. Rates per 100,000; age-adjusted to the 2000 U.S. standard population.

Urban-rural stratification analysis (data available through 2020) revealed broadly consistent patterns—prolonged decline followed by late-2010s increases—with variations in timing and magnitude across county urbanization categories (**Figure 6**; **Table 1**).

Large metropolitan counties demonstrated significant decline during 1999–2014 (APC −1.95%; 95% CI: −2.37 to −1.58) followed by significant increase during 2014–2020 (APC + 5.25%; 95% CI: 3.97 to 7.10). Medium/small metropolitan counties exhibited a similar temporal profile, with significant decline during 1999–2013 (APC −1.82%; 95% CI: −2.51 to −1.24) followed by significant increase during 2013–2020 (APC + 4.99%; 95% CI: 3.60 to 7.07).

Rural counties displayed a more complex multisegment trajectory: non-significant decline during 1999–2005 (APC −1.00%; 95% CI: −1.81 to 1.27), significant decline during 2005–2012 (APC −3.14%; 95% CI: −5.70 to −2.43), non-significant increase during 2012–2018 (APC + 2.91%; 95% CI: −0.75 to 4.12), and sharp, significant increase during 2018–2020 (APC + 8.99%; 95% CI: 4.97 to 11.29).

Annual urbanization category-specific AAMRs (per 100,000) are reported in **Supplementary Table 7**, corroborating these segmented trends and emphasizing the late-2010s increase across all urbanization strata.

### 3.8 State-level variation

**Figure 7:**
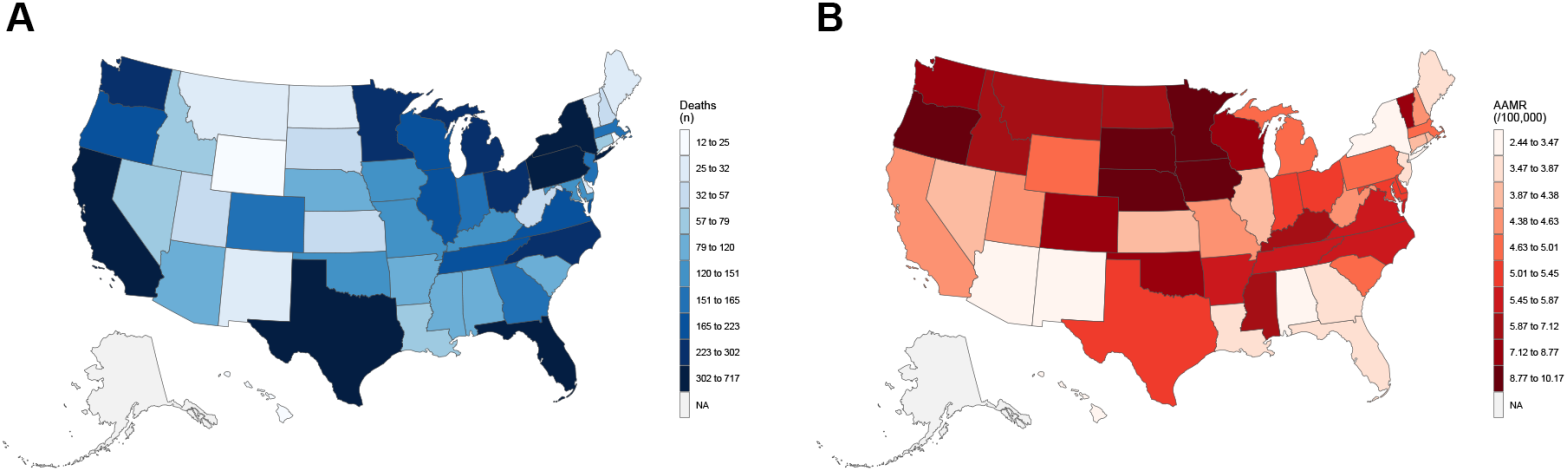
State-level death counts **(A)** and age-adjusted mortality rates **(B)** in adults aged ≥45 years with hematologic malignancy and heart failure, United States, 2023. AAMR, age-adjusted mortality rate. Rates per 100,000; age-adjusted to the 2000 U.S. standard population. States with suppressed counts are labeled NA.

State-level mortality patterns exhibited substantial heterogeneity across jurisdictions. **Figure 7** illustrates 2023 absolute death counts and state-specific AAMRs (per 100,000), with select values suppressed due to low case counts; comprehensive year-by-state data are provided in **Supplementary Table 8**.

Over the entire study period (1999–2023), period-average AAMRs ranged from 2.30 per 100,000 in Arizona to 6.40 per 100,000 in North Dakota (median: 4.33; interquartile range [IQR]: 3.73–5.04). At the national mortality peak in 2021, state-specific AAMRs spanned from 2.34 per 100,000 in Hawaii to 10.39 per 100,000 in Idaho (median: 5.03; IQR: 4.25–7.15).

Temporal comparison between 2013 and 2021 revealed increases in 44 of 46 reporting jurisdictions (median relative change: +48.1%; IQR: +16.3% to +93.9%), with only Alabama and North Dakota experiencing declines. From 2021 to 2023, mortality rates stabilized nationally, with 26 states experiencing decreases and 24 states experiencing increases (median relative change: −0.43%; IQR: −9.09% to +9.90%).

The 2023 death count distribution highlights the concentration of absolute mortality events in more populous states, while the corresponding AAMR map identifies jurisdictions with elevated risk despite lower absolute case numbers. This dual presentation underscores the importance of interpreting both absolute counts and population-adjusted rates to comprehensively assess geographic mortality burden (**Figure 7**; **Supplementary Table 8**).

### 3.9 ARIMA-based mortality forecasting

An ARIMA (3,0,0) model with non-zero mean was fitted to the overall AAMR time series to project future mortality trends. The optimal model specification included autoregressive parameters (AR1: 1.202; AR2: 0.049; AR3: -0.388) and an intercept of 4.030 per 100,000, demonstrating adequate performance with low prediction errors (RMSE = 0.144; MAE = 0.110) and favorable information criteria (AIC = -12.79; BIC = -6.7).

**Figure 8:**
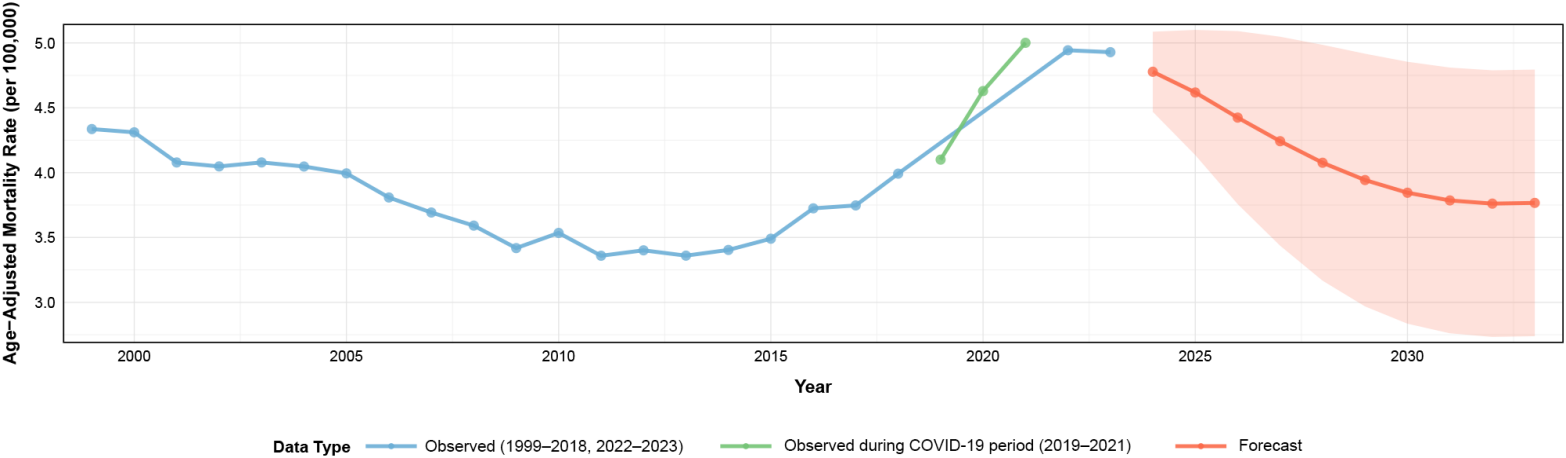
ARIMA-based mortality forecasting for adults aged ≥45 years with hematologic malignancy and heart failure, United States, 1999–2033. ARIMA, Autoregressive Integrated Moving Average; CI, confidence interval. Historical data (1999–2023) shown with observed age-adjusted mortality rates. ARIMA model projections (2024–2033) displayed with 95% confidence intervals. Rates per 100,000; age-adjusted to the 2000 U.S. standard population. The green segment denotes the observed mortality values during the COVID-19 period (2019–2021) within the historical series and does not represent a separate dataset.

Model validation confirmed appropriate specification through residual diagnostics, with the Ljung-Box test indicating no significant autocorrelation in residuals (X² = 17.49, df = 20, *p* = 0.621). Ten-year projections indicated a gradual mortality decline from 4.78 per 100,000 (95% CI: 4.47-5.09) in 2024 to 3.77 per 100,000 (95% CI: 2.74-4.79) by 2033, suggesting potential stabilization following the observed 2018–2021 surge (**Figure 8**; **Supplementary Table 9**).

## 4 Discussion

This comprehensive analysis of 25-year U.S. mortality data provides the first population-based characterization of long-term mortality patterns in adults with concurrent HMs and HF. The analysis reveals a complex temporal trajectory characterized by initial decline through 2013, followed by sustained increases that accelerated markedly during 2018–2021 before stabilizing in recent years.

The sustained mortality decline through 2013 coincided with significant therapeutic advances in both hematologic oncology and HF care. During this period, the introduction of anti-CD20 monoclonal antibodies and early adoption of proteasome inhibitors and immunomodulatory agents improved survival across several HM subtypes. Simultaneously, broader implementation of guideline-directed medical therapy for HF and increased use of device-based interventions contributed to population-level mortality reductions (34–36). The emergence of cardio-oncology as a distinct subspecialty during this period may have promoted systematic surveillance and risk-mitigation strategies (37).

The post-2013 trend reversal may reflect several converging factors. Population aging expanded the at-risk denominator for HM-HF comorbidity (38). Additionally, next-generation targeted therapies introduced novel cardiovascular risks. Systematic reviews report cardiovascular adverse events in approximately 18% of patients receiving carfilzomib (4, 39), while ibrutinib is associated with increased rates of atrial fibrillation and hypertension (40, 41). The temporal alignment between novel agent adoption and mortality trend changes suggests potential relationships, though definitive causal attribution cannot be established from surveillance data.

The sharp 2018–2021 mortality increase likely reflects multiple overlapping influences, with the COVID-19 pandemic representing a significant contributor. Patients with HM experienced elevated COVID-19 case fatality rates (42), while preexisting HF was associated with worse outcomes among COVID-19 patients (43). Healthcare system disruptions during the pandemic led to deferred cancer diagnoses and altered management of chronic conditions, which may have precipitated decompensation in vulnerable patients (44–46). The subsequent stabilization during 2021–2023 may reflect healthcare system adaptation and recovery of care pathways.

This analysis revealed substantial and persistent demographic disparities in HM-HF mortality. Males experienced 85% higher mortality rates than females, reflecting multiple intersecting factors including higher baseline HM incidence rates, particularly for aggressive subtypes requiring intensive treatment, and potential sex-specific differences in cardiovascular susceptibility to treatment-related toxicities (37, 47).

Age-related patterns demonstrated exponentially increasing mortality with advancing age, with adults ≥85 years showing the steepest post-2014 increases. These patterns reflect the convergence of multiple age-related risk factors, including higher baseline cardiovascular comorbidity burden, reduced functional reserve, and complex treatment considerations in older adults (48).

Racial and ethnic disparities in HM-HF mortality reflect longstanding inequities in cancer care, cardiovascular health management, and healthcare access. Non-Hispanic Black and non-Hispanic White populations demonstrated the highest absolute mortality rates, while temporal analysis revealed concerning trends across all racial and ethnic groups. Post-2013 mortality increases were most pronounced among non-Hispanic Black individuals, with a particularly sharp surge during 2017-2021 (APC + 7.88%). These disparities likely reflect multiple systemic factors, including differential access to specialized cardio-oncology care, variations in treatment intensity and adherence, socioeconomic barriers to optimal disease management, and structural inequities within healthcare systems. Non-Hispanic Black patients historically present with more advanced-stage hematologic malignancies and exhibit higher baseline cardiovascular risk profiles, potentially compounding adverse outcomes in the HM-HF population (49, 50). Limited access to clinical trials and newer therapeutic options may further exacerbate disparate outcomes.

Geographic analysis revealed complex urban-rural variations, with rural populations demonstrating particular vulnerability during the late study period. These patterns likely reflect structural challenges in rural healthcare delivery, including limited access to specialized services and greater travel distances to tertiary care centers (51). The COVID-19 pandemic may have exacerbated these disparities through differential healthcare service disruptions (52).

The substantial state-level heterogeneity in mortality rates suggests complex interactions among population demographics, healthcare system characteristics, and regional factors. This geographic variation has important implications for healthcare resource planning and quality improvement initiatives.

Subtype-specific analysis revealed heterogeneous mortality patterns that align closely with the chronology of therapeutic advances in each disease category. MM and CLL demonstrated the earliest trend reversals (2012 and 2014, respectively), corresponding temporally with widespread adoption of novel agents with established cardiovascular toxicity profiles. The MM trajectory particularly reflects the sequential introduction of proteasome inhibitors, immunomodulatory drugs, and monoclonal antibodies, each carrying distinct cardiovascular risk profiles (39). NHL exhibited the most complex multisegment mortality pattern, potentially reflecting the heterogeneity within this disease category and varying adoption rates of targeted therapies across different NHL subtypes. AML demonstrated a later but more pronounced mortality increase (2014–2023 APC + 7.38%), potentially reflecting evolving treatment approaches including hypomethylating agents and targeted therapies such as FLT3 inhibitors (53). Subtype-specific mortality patterns provide insights into the cardiovascular consequences of therapeutic evolution across HMs. The temporal alignment between novel agent introductions and mortality trend changes suggests potential causal relationships; however, the observational nature of mortality surveillance data precludes definitive causal attribution. CML demonstrated an early mortality decline followed by sustained increases, potentially reflecting the therapeutic transition from interferon-based regimens to tyrosine kinase inhibitors, some of which carry established cardiovascular risks (54). These patterns underscore the critical importance of disease-specific cardiovascular risk stratification and monitoring protocols. The varying onset timing and magnitude of mortality increases across disease subtypes suggest that cardiovascular risks may be agent-specific rather than therapeutic class-specific, emphasizing the need for individualized cardio-oncology management approaches based on specific treatment exposures.

The mortality trends documented in this analysis highlight critical gaps in cardio-oncology care capacity. The post-2013 increases coinciding with novel agent adoption suggest inadequate infrastructure to manage the expanding population at risk for treatment-related cardiovascular complications. Priority areas for healthcare system improvement include implementing standardized cardiovascular risk stratification protocols, expanding systematic surveillance programs for treatment-related cardiotoxicity, and strengthening care coordination mechanisms.

The substantial demographic disparities necessitate targeted interventions addressing specific high-risk populations. Enhanced monitoring protocols and intensive cardioprotective strategies may be particularly important for males and older adults (55). Addressing racial and ethnic disparities requires comprehensive interventions including culturally responsive education programs, enhanced patient navigation services, and systematic efforts to eliminate structural healthcare inequities (56).

ARIMA-based forecasting provides insights into future mortality trajectories for adults with concurrent HM and HF. The ARIMA (3,0,0) model demonstrated adequate fit with appropriate residual diagnostics. Projections indicate gradual mortality decline from 4.78 per 100,000 in 2024 to 3.77 per 100,000 by 2033, suggesting stabilization following the 2018–2021 surge. This decline may reflect healthcare system recovery from COVID-19, expanding cardio-oncology infrastructure, and maturing cardiovascular risk management protocols. Increasing adoption of cardioprotective strategies that attenuate therapy-related cardiotoxicity may contribute to this trend.

These forecasts require cautious interpretation. Projections assume continuation of existing patterns and may be altered by novel therapeutics with uncertain cardiovascular toxicity, demographic shifts, or future health emergencies. Widening confidence intervals underscore inherent long-term uncertainty. The predicted decline contrasts paradoxically with the expanding at-risk population due to aging demographics and improved cancer survival. This suggests systematic cardiovascular risk management may offset anticipated disease burden increases through proactive cardio-oncology interventions. These forecasts have important implications for healthcare planning and resource allocation. Projected stabilization indicates recent cardio-oncology investments may yield population-level benefits. However, continued surveillance, model refinement, and ensemble forecasting approaches remain essential to validate projections and identify deviations requiring intervention.

### 4.1 Limitations

This analysis has several important limitations. Multiple cause-of-death data derived from death certificates possess inherent accuracy limitations and potential for misclassification (57). The requirement for concurrent documentation of both conditions may lead to systematic underestimation of the true population burden. Critical clinical information including disease stage, specific treatment regimens, and individual risk factor profiles remains unavailable in mortality surveillance data, limiting detailed risk stratification.

While temporal correlations between treatment innovations and mortality trends suggest potential relationships, the observational study design precludes definitive causal attribution. Surveillance bias resulting from increased clinical awareness of treatment-related cardiovascular toxicity may have enhanced recognition and documentation of cardiovascular causes of death in cancer patients. Additionally, the COVID-19 pandemic represents a unique confounder that complicates interpretation of recent mortality trends through direct viral effects, healthcare system disruptions, and altered care-seeking behaviors (58).

The mortality patterns documented highlight critical research priorities in cardio-oncology. Future investigations should focus on agent-specific cardiotoxicity mechanisms, with particular attention to apparent sex-based differences in cardiovascular susceptibility. Predictive biomarker development represents an urgent priority, with current evidence most strongly supporting serial high-sensitivity cardiac troponin and natriuretic peptide monitoring for early detection and risk-adapted cardioprotection (59).

Randomized controlled trials evaluating prophylactic cardioprotective interventions, including ACE inhibitors, beta-blockers, and novel agents such as SGLT2 inhibitors, are essential for establishing optimal prevention approaches in HM populations. Comparative effectiveness research should evaluate different surveillance strategies to determine optimal monitoring approaches while balancing early detection benefits with patient burden and cost-effectiveness considerations (60).

## 5 Conclusions

This 25-year analysis reveals complex HM-HF mortality patterns: decline through 2013, increases during 2018–2021, and recent stabilization. ARIMA modeling projects gradual decline through 2033, suggesting stabilization following healthcare recovery, though forecasting uncertainties warrant caution. Substantial demographic and geographic disparities persist, with projected decline contrasting with expanding at-risk populations due to aging and improved cancer survival. Systematic cardiovascular risk management in hematologic malignancy patients represents essential priorities for clinical practice and policy development.

## Data Availability

The original contributions presented in the study are included in the article/**Supplementary Material**. Further inquiries can be directed to the corresponding author.
