# Supplementary material for: Temporal trends, disparities, and ARIMA forecasts of mortality among U.S. adults with coexisting hematologic malignancy and heart failure, 1999–2023, with projections to 2033
Source: Front Oncol. 2026 Apr 22;16:1818278. doi: 10.3389/fonc.2026.1818278 (PMC13143579; doi:10.3389/fonc.2026.1818278)
Supplement: Supplementary file 10 [file Table10.docx]

**Supplementary File S1**

# R code for ARIMA-based forecasting of mortality rates

# Load packages

library(data.table)

library(dplyr)

library(tidyverse)

library(ggpubr)

library(ggtext)

library(ggplot2)

library(forecast)

library(tseries)

# Set working directory

setwd("C:/Users/yue/Desktop/CDC/")

# Read input data

finaldata <- fread("joinpint_data/finaldata.csv")

# Create a classification variable

finaldata$Classification_variable <- paste(finaldata$Classification, finaldata$variable, sep = "_")

# Select the series for forecasting

# In this dataset, "Sex_Both" corresponds to the overall series used for forecasting.

# Other subgroups can be analyzed by changing the value inside the brackets.

data <- finaldata[finaldata$Classification_variable == "Sex_Both"]

# Display selected data

print(data)

# Convert mortality rate to a time series object

AAMR_ts <- ts(data$AAMR, start = c(min(data$Year)), frequency = 1)

# Plot the original time series

plot(AAMR_ts, main = "AAMR Rate Over Time", ylab = "AAMR Rate", xlab = "Year")

# Check stationarity using the Augmented Dickey-Fuller test

adf.test(AAMR_ts)

# If the p value is > 0.05, the series may be non-stationary and differencing can be explored.

# First-order differencing

first_AAMR_ts <- diff(AAMR_ts)

adf.test(first_AAMR_ts)

# Second-order differencing

second_AAMR_ts <- diff(first_AAMR_ts)

adf.test(second_AAMR_ts)

# Inspect ACF and PACF after differencing

acf(second_AAMR_ts)

pacf(second_AAMR_ts)

# Exploratory differencing and ACF/PACF inspection were performed as part of preliminary

# time-series assessment. The final reported model was selected using auto.arima()

# on the original annual AAMR series.

# Fit ARIMA model using automatic model selection

arima_model <- auto.arima(AAMR_ts)

# Model summary

summary(arima_model)

# Print selected ARIMA order and model coefficients

cat("Selected ARIMA order (p,d,q):", paste(arimaorder(arima_model), collapse = ","), "\n")

print(arima_model$coef)

# Extract model performance metrics

rmse <- sqrt(mean(residuals(arima_model)^2, na.rm = TRUE))

mae <- mean(abs(residuals(arima_model)), na.rm = TRUE)

aic_value <- AIC(arima_model)

bic_value <- BIC(arima_model)

cat("RMSE:", round(rmse, 3), "\n")

cat("MAE:", round(mae, 3), "\n")

cat("AIC:", round(aic_value, 2), "\n")

cat("BIC:", round(bic_value, 2), "\n")

# Check residual autocorrelation

acf(residuals(arima_model))

# Ljung-Box test for residual autocorrelation

ljung_box <- Box.test(residuals(arima_model), lag = 20, type = "Ljung-Box")

print(ljung_box)

# Forecast mortality rates for the next 10 years

forecasted_values <- forecast(arima_model, h = 10)

# Print forecast results

print(forecasted_values)

cat("Forecast for 2024:", round(as.numeric(forecasted_values$mean[1]), 2), "\n")

cat("Forecast for 2033:", round(as.numeric(tail(forecasted_values$mean, 1)), 2), "\n")

# Basic forecast plot

plot(forecasted_values)

# Convert data to a data frame for ggplot

forecast_df <- data.frame(

Year = c(time(AAMR_ts), time(forecasted_values$mean)),

AAMRRate = c(AAMR_ts, forecasted_values$mean),

Type = c(rep("Observed", length(AAMR_ts)), rep("Forecast", length(forecasted_values$mean)))

)

# Distinguish the observed COVID-19 period (2019-2021) within the historical series

forecast_df$Type <- ifelse(

forecast_df$Year %in% 2019:2021 & forecast_df$Type == "Observed",

"Observed during COVID-19 period (2019–2021)",

forecast_df$Type

)

forecast_df$Type <- ifelse(

forecast_df$Type == "Observed",

"Observed (1999–2018, 2022–2023)",

forecast_df$Type

)

# Plot mortality trends and forecast in a publication-style figure

p1 <- ggplot(forecast_df, aes(x = Year, y = AAMRRate, color = Type)) +

geom_line(size = 1.2, alpha = 0.9) +

geom_point(size = 2.5, alpha = 0.8) +

scale_color_manual(

values = c(

"Observed (1999–2018, 2022–2023)" = "#6BAED6",

"Observed during COVID-19 period (2019–2021)" = "#74C476",

"Forecast" = "#FB6A4A"

),

breaks = c(

"Observed (1999–2018, 2022–2023)",

"Observed during COVID-19 period (2019–2021)",

"Forecast"

),

labels = c(

"Observed (1999–2018, 2022–2023)",

"Observed during COVID-19 period (2019–2021)",

"Forecast"

)

) +

labs(

title = "Age-Adjusted Mortality Rate Over Time",

x = "Year",

y = "Age-Adjusted Mortality Rate (per 100,000)",

color = "Data Type"

) +

theme_minimal() +

theme(

panel.background = element_rect(fill = "white", color = NA),

panel.grid.major = element_line(color = "#E5E5E5", size = 0.3),

panel.grid.minor = element_line(color = "#F0F0F0", size = 0.2),

panel.border = element_rect(color = "black", fill = NA, size = 0.8),

plot.title = element_text(hjust = 0.5, size = 14, face = "bold", margin = margin(b = 20)),

axis.title.x = element_text(size = 12, face = "bold", margin = margin(t = 10)),

axis.title.y = element_text(size = 12, face = "bold", margin = margin(r = 10)),

axis.text = element_text(size = 10, color = "black"),

axis.text.x = element_text(angle = 0),

legend.position = "bottom",

legend.title = element_text(size = 11, face = "bold"),

legend.text = element_text(size = 10),

legend.background = element_blank(),

legend.box.background = element_blank(),

legend.key = element_blank(),

legend.margin = margin(t = 15),

legend.spacing.x = unit(0.5, "cm"),

plot.margin = margin(20, 20, 20, 20)

) +

scale_x_continuous(

breaks = seq(ceiling(min(data$Year) / 5) * 5,

ceiling((max(data$Year) + 10) / 5) * 5, by = 5),

expand = c(0.02, 0)

) +

scale_y_continuous(

breaks = scales::pretty_breaks(n = 8),

expand = c(0.02, 0)

)

print(p1)

# Create a data frame with 95% confidence intervals

forecast_with_ci <- data.frame(

Year = time(forecasted_values$mean),

Mean = as.numeric(forecasted_values$mean),

Lower = as.numeric(forecasted_values$lower[, 2]),

Upper = as.numeric(forecasted_values$upper[, 2])

)

# Create complete data for plotting

forecast_complete_df <- rbind(

subset(forecast_df, Type != "Forecast"),

data.frame(

Year = forecast_with_ci$Year,

AAMRRate = forecast_with_ci$Mean,

Type = "Forecast"

)

)

# Plot with forecast confidence interval

p2 <- ggplot() +

geom_line(data = forecast_complete_df,

aes(x = Year, y = AAMRRate, color = Type),

size = 1.2, alpha = 0.9) +

geom_point(data = forecast_complete_df,

aes(x = Year, y = AAMRRate, color = Type),

size = 2.5, alpha = 0.8) +

geom_ribbon(data = forecast_with_ci,

aes(x = Year, ymin = Lower, ymax = Upper),

fill = "#FB6A4A", alpha = 0.2) +

scale_color_manual(

name = "Data Type",

values = c(

"Observed (1999–2018, 2022–2023)" = "#6BAED6",

"Observed during COVID-19 period (2019–2021)" = "#74C476",

"Forecast" = "#FB6A4A"

),

breaks = c(

"Observed (1999–2018, 2022–2023)",

"Observed during COVID-19 period (2019–2021)",

"Forecast"

),

labels = c(

"Observed (1999–2018, 2022–2023)",

"Observed during COVID-19 period (2019–2021)",

"Forecast"

)

) +

labs(

title = "Age-Adjusted Mortality Rate Over Time with Forecast",

x = "Year",

y = "Age-Adjusted Mortality Rate (per 100,000)"

) +

theme_minimal() +

theme(

panel.background = element_rect(fill = "white", color = NA),

panel.grid.major = element_line(color = "#E5E5E5", size = 0.3),

panel.grid.minor = element_line(color = "#F0F0F0", size = 0.2),

panel.border = element_rect(color = "black", fill = NA, size = 0.8),

plot.title = element_text(hjust = 0.5, size = 14, face = "bold", margin = margin(b = 20)),

axis.title.x = element_text(size = 12, face = "bold", margin = margin(t = 10)),

axis.title.y = element_text(size = 12, face = "bold", margin = margin(r = 10)),

axis.text = element_text(size = 10, color = "black"),

legend.position = "bottom",

legend.title = element_text(size = 11, face = "bold"),

legend.text = element_text(size = 10),

legend.background = element_blank(),

legend.box.background = element_blank(),

legend.key = element_blank(),

legend.margin = margin(t = 15),

legend.spacing.x = unit(0.5, "cm"),

legend.direction = "horizontal",

plot.margin = margin(20, 20, 20, 20)

) +

scale_x_continuous(

breaks = seq(ceiling(min(data$Year) / 5) * 5,

ceiling((max(data$Year) + 10) / 5) * 5, by = 5),

expand = c(0.02, 0)

) +

scale_y_continuous(

breaks = scales::pretty_breaks(n = 8),

expand = c(0.02, 0)

) +

guides(color = guide_legend(

title = "Data Type",

keywidth = unit(1.5, "cm"),

keyheight = unit(0.5, "cm"),

override.aes = list(size = 1.2, alpha = 0.9)

))

print(p2)

# Alternative simplified plotting approach

p3 <- ggplot() +

geom_line(data = subset(forecast_df, Type != "Forecast"),

aes(x = Year, y = AAMRRate, color = Type),

size = 1.2, alpha = 0.9) +

geom_point(data = subset(forecast_df, Type != "Forecast"),

aes(x = Year, y = AAMRRate, color = Type),

size = 2.5, alpha = 0.8) +

geom_ribbon(data = forecast_with_ci,

aes(x = Year, ymin = Lower, ymax = Upper),

fill = "#FB6A4A", alpha = 0.2) +

geom_line(data = forecast_with_ci,

aes(x = Year, y = Mean),

color = "#FB6A4A", size = 1.2, alpha = 0.9) +

geom_point(data = forecast_with_ci,

aes(x = Year, y = Mean),

color = "#FB6A4A", size = 2.5, alpha = 0.8) +

scale_color_manual(

name = "Data Type",

values = c(

"Observed (1999–2018, 2022–2023)" = "#6BAED6",

"Observed during COVID-19 period (2019–2021)" = "#74C476"

),

breaks = c(

"Observed (1999–2018, 2022–2023)",

"Observed during COVID-19 period (2019–2021)"

),

labels = c(

"Observed (1999–2018, 2022–2023)",

"Observed during COVID-19 period (2019–2021)"

)

) +

annotate("text", x = Inf, y = -Inf,

label = "■ Forecast",

hjust = 1.1, vjust = -0.5,

color = "#FB6A4A", size = 3.5) +

labs(

title = "Age-Adjusted Mortality Rate Over Time with Forecast",

x = "Year",

y = "Age-Adjusted Mortality Rate (per 100,000)"

) +

theme_minimal() +

theme(

panel.background = element_rect(fill = "white", color = NA),

panel.grid.major = element_line(color = "#E5E5E5", size = 0.3),

panel.grid.minor = element_line(color = "#F0F0F0", size = 0.2),

panel.border = element_rect(color = "black", fill = NA, size = 0.8),

plot.title = element_text(hjust = 0.5, size = 14, face = "bold", margin = margin(b = 20)),

axis.title.x = element_text(size = 12, face = "bold", margin = margin(t = 10)),

axis.title.y = element_text(size = 12, face = "bold", margin = margin(r = 10)),

axis.text = element_text(size = 10, color = "black"),

legend.position = "bottom",

legend.title = element_text(size = 11, face = "bold"),

legend.text = element_text(size = 10),

legend.background = element_blank(),

legend.box.background = element_blank(),

legend.key = element_blank(),

legend.margin = margin(t = 15),

legend.spacing.x = unit(0.5, "cm"),

legend.direction = "horizontal",

plot.margin = margin(20, 20, 20, 20)

) +

scale_x_continuous(

breaks = seq(ceiling(min(data$Year) / 5) * 5,

ceiling((max(data$Year) + 10) / 5) * 5, by = 5),

expand = c(0.02, 0)

) +

scale_y_continuous(

breaks = scales::pretty_breaks(n = 8),

expand = c(0.02, 0)

)

print(p3)
